# Comorbidity Profiling in Rural and Urban Population of West Bengal, India: Report From a Community-Based Primary Healthcare System

**DOI:** 10.7759/cureus.51436

**Published:** 2024-01-01

**Authors:** Deyashini Mukherjee, Subhabrata Moitra, Punyabrata Gun, Mrinmoy Bera, Piyali Dey-Biswas, Rahul Mukherjee

**Affiliations:** 1 Cardiology, University Hospitals Coventry and Warwickshire, Coventry, GBR; 2 Medicine and Dentistry, University of Alberta, Edmonton, CAN; 3 General Medicine, Swasthya Shiksha Nirman (Rational Medicine Network), Kolkata, IND; 4 Epidemiology and Public Health, Swasthya Shiksha Nirman (Rational Medicine Network), Kolkata, IND; 5 Respiratory Medicine and Physiology, Birmingham Heartlands Hospital, Birmingham, GBR

**Keywords:** demographic transition, population health, non-communicable disease, comorbidity, primary healthcare

## Abstract

Introduction

The burden of non-communicable diseases (NCDs) is fast changing across the world, especially in the context of rapid urbanization, adoption of Western lifestyles, and an aging multi-morbid population. Over the last three decades, India has undergone a significant demographic and socioeconomic transition. For effective targeting of health system resources and services, it is essential to understand how the prevalence of NCDs varies among population groups across India. We set out to understand the distribution of NCDs and co-morbidities in urban and rural West Bengal.

Methods

As part of a service improvement project, data was collected from four urban and four rural community-based clinics across West Bengal, India. The reason for visiting the healthcare center was recorded as the primary diagnosis and co-morbidities were recorded per the Elixhauser comorbidity scoring criteria. Associations between all the demographic variables and NCDs were studied using the Poisson regression model and multivariate analysis. Demographic profile, co-morbidities, and Elixhauser comorbidity index were expressed as frequency (%), mean (standard deviation, SD), or median (interquartile range, IQR) as appropriate.

Results

We obtained data from 1244 patients of which 886 (71%) were from urban areas and 358 (29%) were from rural areas. Patients were mostly female (61%) and had a mean (SD) age of 53 (11) years. There was a positive correlation between living in an urban residence and age, body mass index (BMI), hypertension, cardiovascular disease (CVD), and respiratory disease. There was a positive correlation between CVD and age, male sex, living in an urban residence, and hypertension but did not correlate positively with diabetes. BMI positively correlated with living in an urban residence, hypertension, diabetes, and musculoskeletal disorders. We observed a significantly higher prevalence of musculoskeletal (p=0.002) and psychological diseases (p<0.001) in the rural population, while the prevalence of hypertension (p<0.001) and respiratory diseases among the participants living in urban areas was higher (p<0.001). There was no statistically significant difference in the prevalence of diabetes between urban and rural areas (p=0.38). In the multivariable analyses, we observed that increased age, being overweight, and living in urban areas were associated with hypertension (prevalence ratio (PR): 1.40, 1.30, and 1.30, respectively; all p-values <0.05). An interaction between sex and living area was associated with a lower prevalence of musculoskeletal diseases (PR: 0.34; 95%CI: 0.18-0.66), i.e., musculoskeletal diseases were less prevalent in males living in urban areas (p=0.002).

Conclusion

There is a rise in multimorbidity with changing demographic patterns and a narrowing of the urban-rural gap in disease distribution. More investment is required in risk factor prevention, screening, and treatment, with greater accessibility of healthcare resources for those in rural areas. Further work needs to be done to study the trends and distribution of NCDs in West Bengal to inform healthcare policy.

## Introduction

In 2015, as part of Sustainable Development Goal 3, the United Nations (UN) member states set the target of reducing premature mortality from non-communicable diseases (NCDs) by one-third by 2030. Given India’s huge population, its achievements are critical to reaching these global targets [1]. For effective targeting of health system resources and services, it is essential to understand how the prevalence of NCDs varies among population groups across this vast country. Given the predisposition of people of South Asian ethnicity toward cardiometabolic diseases, it is particularly important to assess the distribution of cardiometabolic diseases. India has witnessed a rapid change in morbidity patterns in NCDs in the past 30 years where the estimated proportion of disability-adjusted life-years (DALYs) attributable to NCDs has risen from 31% of total DALYs in 1990 to 55% in 2016 [2]. Of note, the global burden of cardiovascular disease (CVD) in terms of DALYs went from being the third highest category in 1990 to the highest category in 2019, thereby overtaking respiratory infections including tuberculosis and neonatal and maternal disorders [2]. Furthermore, over the same time interval, the global burden of neoplasms in terms of DALYs went from being the sixth highest to second highest [2]. In 2019, CVD accounted for 24.21% of total DALYs, with over one-third of this being attributable to hypertension [2]. When focusing on India, the highest contributor to DALY is CVD, followed by maternal and neonatal disorders, respiratory infections, and tuberculosis [2]. Of the various behavioral, environmental, occupational, and metabolic risks or clusters of risks, metabolic risks warrant particular policy attention, due to their large contribution to global disease burden, increasing trends, and variable patterns across countries at the same level of development [2,3]. The higher proportion of DALYs due to NCDs is due to population aging and population growth [3]. The increasing proportion of DALYs attributable to NCDs calls for a more detailed understanding of their distribution to improve health, risk stratification, and modification.

As India is a large and diverse country with significant regional variation, it is important to understand the prevalence of NCDs at a state-level detail to drive healthcare policy regarding targeted prevention, screening, and treatment services. The Epidemiology of Non-Communicable Diseases in Rural Areas (ENDIRA) study compared the prevalence of CVDs in rural areas in the Indian state of Kerala [4]. They found that adults below the poverty line were less likely to have diabetes, dyslipidemia, and hypertension. Interestingly, adults below the poverty line were more likely to have respiratory diseases and stroke but not myocardial infarction or cancer [4].

In this study, we aimed to assess the intra-state prevalence and urban-rural distribution of NCDs in the state of West Bengal, India. We created a database of comorbidities covering all patients who attended the eight low-cost community-based clinics run by a voluntary organization Swasthya Shiksha Nirman.

## Materials and methods

A service improvement project aimed at improving data quality was undertaken as a systematic, data-guided activity designed to bring about improvements in health delivery in our network’s community-based primary healthcare system by assessing the distribution of NCDs in West Bengal. Data was prospectively and routinely collected as part of this service improvement project to improve comorbidity data completeness, whereby a standard history-taking proforma was designed and implemented in all eight of Swasthya Shiksha Nirman's low-cost community-based clinics spread across the state of West Bengal, India. The four urban clinics were based in Kolkata (Dattabad), Jalpaiguri, Barasat, and Halisahar. The four rural clinics were in Chengail, Debra, Mathabhanga, and Chandipur. The project did not use any intervention and the same proforma was used on all patients in all of the community-based clinics. All data collectors were trained in using the proforma based on the Elixhauser comorbidity scoring criteria [5]. The project was approved by the executive board of Swasthya Siksha Nirman.

All adult patients (≥18 years) attending eight low-cost outpatient clinics of Swasthya Shiksha Nirman (part of the Rational Medicine Network) in West Bengal, India, between 1st May 2019 and 31st July 2019 were recruited in this service improvement project. The study included all visits, which were a mixture of new and follow-up visits. The reason for the clinic attendance on the day, be it a new or follow-up attendance, was recorded as the primary diagnosis and comorbidities were recorded as per the Elixhauser comorbidity scoring criteria [5]. Standard patient demographics (age, height, and weight) were also collected. We obtained data from 1244 patients.

Demographic profile (age, sex, and body mass index (BMI)), comorbidities, and Elixhauser comorbidity index were expressed as frequency (%), mean (standard deviation (SD)), or median (interquartile range (IQR)) as appropriate. We performed Poisson regression models and multivariate analysis to test the associations between all the demographic variables (age, sex, BMI, area, and an interaction between sex and domicile) and each of the comorbidities. We used age as decile (per 10 years) and BMI as binary (normal weight vs. overweight/obese) categorical variables in the regression models and the results were presented as prevalence ratio (PR) and 95% confidence interval (CI). All statistical analysis was performed using Stata version 17.

## Results

We obtained data from 1244 patients of which 886 (71%) were from urban areas and 358 (29%) were from rural areas. Patients were mostly female (61%) and had a mean (SD) age of 53 (11) years. Please see Table 1 for a full breakdown of the demographic profile of all study participants.

**Table 1:** Demographic characteristics of study participants Data is shown as mean (SD), median (IQR), or frequency (%) unless otherwise specified. BMI, body mass index; SD, standard deviation; IQR, interquartile range; CVD, cardiovascular diseases

	N=1244
Age (years), mean (SD)	53 (11)
Sex (female), n (%)	755 (61)
BMI (kg/m^2^), mean (SD)	23.2 (4.6)
Domicile, n (%)	
Rural	711 (57)
Urban	532 (43)
Elixhauser score, median (IQR)	2 (1-3)
Diseases, n (%)	
Hypertension	316 (25)
Diabetes	250 (20)
Musculoskeletal problems	219 (17)
CVD	146 (12)
Gastrointestinal disorders	115 (9)
Psychological conditions	77 (6)
Respiratory diseases	47 (4)
Thyroidal problems	53 (4)
Other conditions	95 (8)

**Figure 1:**
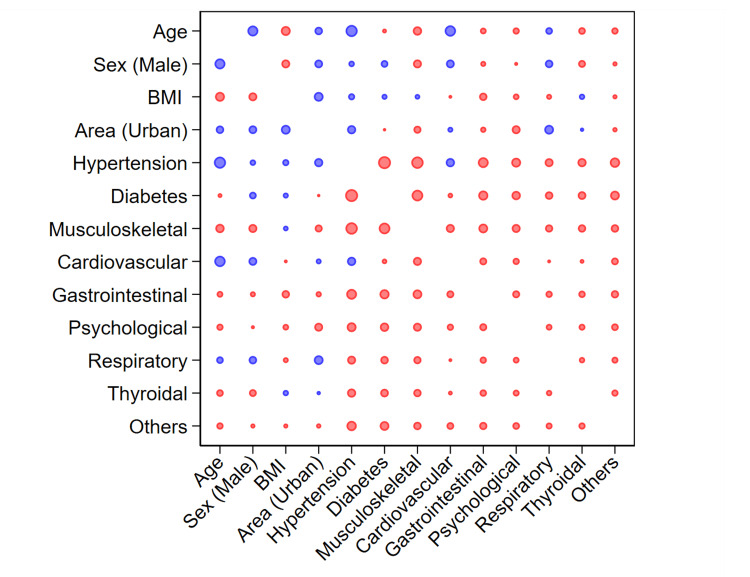
Correlation bubble plot showing the associations between demographic characteristics and comorbidities Blue color indicates a positive correlation and red color indicates a negative correlation. The size of the bubbles indicates the correlation coefficient.

Figure 1 shows the correlation between different variables. There was a positive correlation between living in an urban residence and age, BMI, hypertension, CVD, and respiratory disease. There was a positive correlation between CVD and age, male sex, living in an urban residence, and hypertension but did not correlate positively with diabetes. BMI positively correlated with living in an urban residence, hypertension, diabetes, and musculoskeletal disorders.

**Figure 2:**
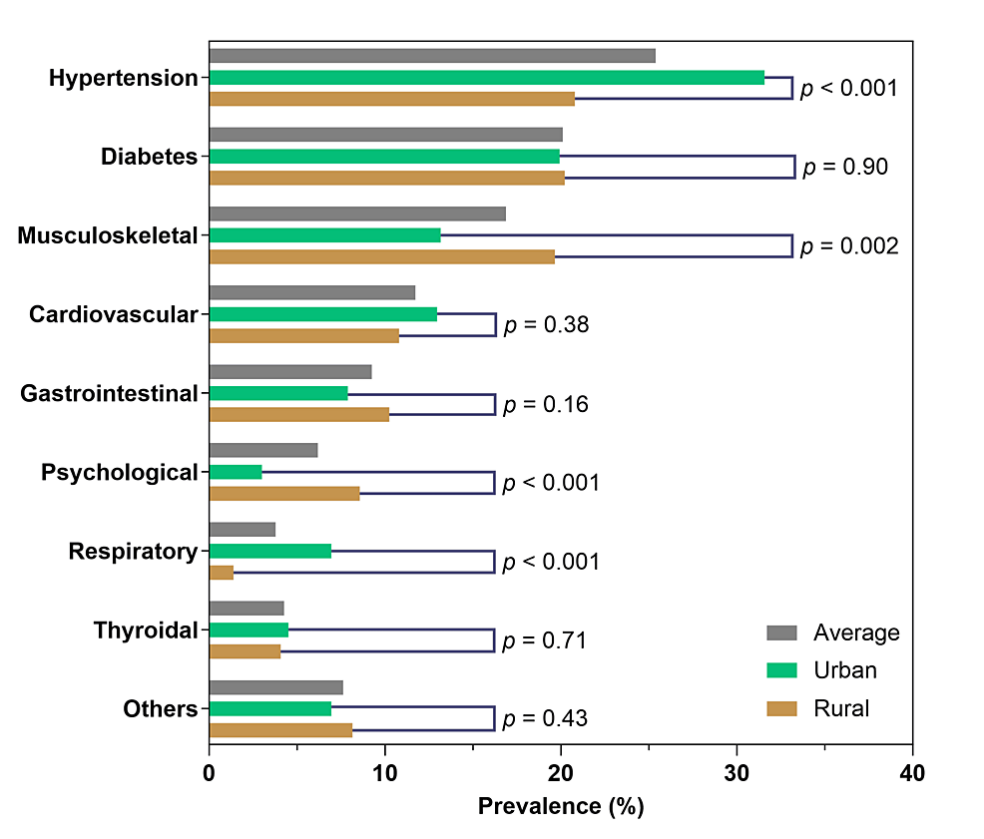
Bar diagram of the comorbidities in the total population and population subgroup by area P-values were obtained from chi-squared tests between urban and rural populations

Figure 2 demonstrates the differences in the distribution of different variables. Patients living in urban areas were more overweight/obese (43 vs. 55%, chi-squared p<0.001) than their rural counterparts. Hypertension and diabetes were the most prevalent comorbidities (25 and 20%, respectively) followed by musculoskeletal (17%) and CVD (12%). Hypertension was significantly more common in urban areas (p<0.001). There was a higher prevalence of CVD in urban areas but this was not statistically significant (p=0.38). There was no significant difference in the prevalence of diabetes between urban and rural areas (p=0.90). We observed a significantly higher prevalence of musculoskeletal (p=0.002) and psychological diseases (p<0.001) in the rural population, while the prevalence of respiratory diseases among the participants living in urban areas was higher (p<0.001).

**Figure 3:**
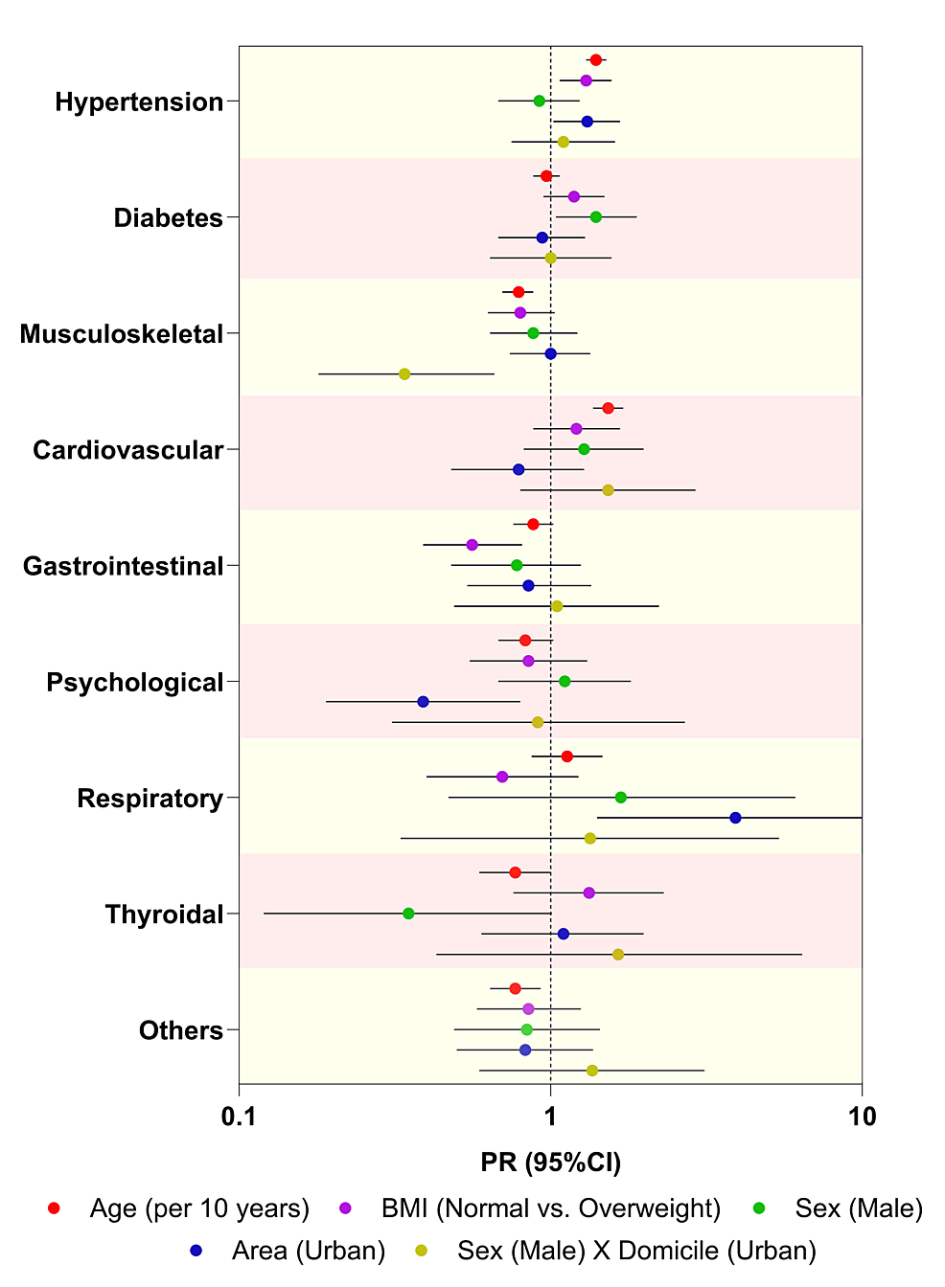
Associations between demographic characteristics and comorbidities Data is shown as PR (colored circle symbol) and 95% CI (horizontal error bars). Age and BMI were considered as categorical variables. See methods for details. PR, prevalence ratio; CI, confidence interval

In the multivariable analyses, we observed that increased age, being overweight, and living in urban areas were associated with hypertension (PRs: 1.40, 1.30, and 1.30, respectively; all p-values <0.05). An interaction between sex and living area was associated with lower prevalence of musculoskeletal diseases (PR: 0.34; 95%CI: 0.18-0.66), i.e., musculoskeletal diseases were less prevalent in males living in urban areas. While living in urban areas was associated with a lower prevalence of psychological diseases (PR: 0.39; 95%CI: 0.19-0.79), it was associated with a higher prevalence of respiratory diseases (PR: 3.92; 95%CI: 1.41-10.90) (Figure 3).

## Discussion

While hypertension is the most common health condition among people seeking primary healthcare in West Bengal, diabetes and musculoskeletal disorders are not far behind. This study showed that hypertension was more common with increasing age, male sex, urban residence, and CVD. This is in keeping with Geldzetser’s extensive work, which showed that high blood glucose and high systolic blood pressure were common among poor individuals in middle and old age and smoking was most prevalent among men, in rural areas and poorer wealth quintiles [6,7].

The prevalence of diabetes and hypertension was higher in middle and old age across all geographical areas and sociodemographic groups in India, and the prevalence of hypertension among young adults was higher than previously thought [7]. They also crucially identified important variations in risk among states and by individuals’ sociodemographic characteristics [7]. Similarly, another report suggested that nearly 50% of Indians aged 45 and above suffer from metabolic conditions and the rate is higher in urban populations than their rural counterpart [8]. Harikrishnan et al. found that overweight or obese BMI, female sex, and urban residence were strongly associated with metabolic syndrome, which correlated with coronary artery disease [9]. It is particularly concerning given that South Asian adults are thought to be predisposed to developing CVD as a result of a combination of innate, cultural, and lifestyle factors [10]. Our findings showed that when looking at trends there was no statistically significant difference in CVD risk or diabetes between the urban and rural areas, which is also in keeping with findings in the study by Wetzel et al. [11].

This study found a trend of higher CVD in urban areas, although not statistically significant. This could possibly be associated with a diet containing high quantities of salt and calorie-dense foods, as well as potentially more sedentary lifestyles with less physical activity. However, Ranasinghe et al.'s systematic review showed a narrower gap in dietary habits between rural and urban areas; therefore, this does not fully explain the difference observed [12]. More work needs to be done to compare the dietary habits and physical activity of the population in urban and rural areas in West Bengal, India.

This study found a high prevalence of multimorbidity among adults in the state of West Bengal, India. This is consistent with the findings of Prenissl et al., which highlight the need to avoid single-disease-focused vertical programs in South Asia and move toward a more integrated approach to manage the ever-growing multimorbid patients [13].

This study did not find any significant difference in the prevalence of diabetes between the rural and urban areas. This is consistent with the summary of findings of Ranasinghe et al.’s systematic review, which showed a relatively high burden of diabetes and pre-diabetes across several states in India, with a transition toward a narrower urban-rural gap due to a myriad of factors, for example, narrower gap in living standards, more calorie or fat-dense foods in place of cereals, and increase in sedentary habits due to mobile phones and television [12]. In Kerala, there was a trend toward more women from rural areas presenting to an endocrinology center with diabetes [14]. Studies across the world have suggested a U-shaped relationship between birthweight and risk of type 2 diabetes later in life [15,16]. In our project, there could have been an under-reporting of diabetes, particularly in rural areas. Corsi and Subramanian found a positive correlation between higher quintiles of wealth and self-reporting of diabetes, which poses further challenges in addressing the burden of diabetes given the high proportion of people living in extreme poverty [17]. More work needs to be done to look at the trends of the prevalence of diabetes in West Bengal and compare the distribution in urban versus rural areas, as well as explore the association of diabetes with birthweight.

Musculoskeletal disease was significantly higher in rural areas. This is in keeping with the increased prevalence of manual work, leading to increased degenerative musculoskeletal diseases such as back and knee pain.

There was a significantly higher prevalence of psychological disorders in rural areas. This may in part be attributable to farmer suicides, which were at their record high nationally each year between 2019 and 2021 [18]. Kallakuri et al. found that anxiety, depression, and suicidal ideation were common in the rural population of Andhra Pradesh, with stress due to financial loss having a significant impact [19]. Further work needs to be done to evaluate the nature of psychological disease in rural areas and adapt better support systems and accessibility for them to access mental health support.

This study found that respiratory disease was more strongly associated with residence in urban areas. This is not surprising, given the high levels of air pollution in urban areas. Further work needs to be done to explore the link between air pollution, respiratory disease, and CVD, given that air pollution is the leading environmental risk factor for CVD in India and worldwide [2,20]. 

While the higher prevalence of NCDs in urban areas could be explained by the social, environmental, and cultural changes associated with India’s economic transition from a pre-industrial agrarian economy to industrialization, it might also be due to the under-reporting in rural areas as there has been a greater disparity in healthcare distribution between rural and urban areas. This disparity is one of the major leading causes of underdiagnosis of chronic diseases, which leads to a concomitant economic burden of diseases [21]. Thus, a rational approach to the diagnosis and management of comorbidities should be amended in India to prioritize a generalized healthcare system that would cater to both rural and urban communities equally.

Our project has some limitations. First, this is a clinic-based study like Sridhar et al. and not a population-based study [14]. Therefore it relied on the healthcare utilization patterns of the respective populations. Although the eight clinics were spread out across the state of West Bengal, this is a snapshot rather than a true representation of the urban-rural distribution of NCDs in the state. Hence there is a risk of a degree of selection bias. Second, no interventions were made as it was a data quality improvement project, which meant that confirmatory tests such as blood tests for diabetes were not always available and relied on inferring the diagnosis from the patient’s drug history. Third, the study does not record precise socioeconomic status. It captures the comorbidities of people utilizing these voluntary sector clinics. In the Indian context, this means they represent the lower middle class to those below the poverty line who cannot access high-cost corporate healthcare. However, these socioeconomic classes constitute more than two-thirds of the population of West Bengal based on the census reports.

Future work should aim to study the population overall and take more detailed information about the following: socioeconomic status (e.g., wealth quintile), dietary habits, activity levels, etc. Further work should also look at the interventions or treatments being initiated or continued to see if there are any urban-rural differences in health outcomes. This will inform future healthcare policy aimed at integrated management of multiple NCDs.

## Conclusions

This survey shows that multimorbidity is on the rise in the state of West Bengal, India and that the urban-rural distribution of disease is changing in the context of an evolving demographic profile, particularly with respect to CVD and diabetes. More investment is required in risk factor prevention, screening, and treatment, with greater accessibility of healthcare resources for those in rural areas. More work needs to be done, particularly in West Bengal, to study the trends and distribution of NCDs.
